# Primary Mediastinal Germ Cell Tumors—The University of Western Ontario Experience

**DOI:** 10.3390/curroncol28010010

**Published:** 2020-12-08

**Authors:** Arnon Lavi, Eric Winquist, Shiva M. Nair, Joseph L. Chin, Jonathan Izawa, Ricardo Fernandes, Scott Ernst, Nicholas E. Power

**Affiliations:** 1 Department of Surgery, Urology Division, Schulich School of Medicine and Dentistry, University of Western Ontario, London, ON N6A 5W9, Canada; arnon.lavi@lhsc.on.ca (A.L.); Eric.Winquist@lhsc.on.ca (E.W.); nair.shiva@gmail.com (S.M.N.); joseph.chin@lhsc.on.ca (J.L.C.); jonathan.izawa@lhsc.on.ca (J.I.); 2 Department of Oncology, Division of Medical Oncology, Schulich School of Medicine and Dentistry, University of Western Ontario, London, ON N6A 4L6, Canada; ricardo.fernandes@lhsc.on.ca (R.F.); scott.ernst@lhsc.on.ca (S.E.)

**Keywords:** testicular cancer, extragonadal germ cell tumor, primary mediastinal germ cell tumor

## Abstract

Extragonadal germ cell tumors account for 2–5.7% of germ cell tumors (GCTs). Of these, primary mediastinal GCTs (PMGCTs) are responsible for 16–36% of cases. Given the rarity of these tumors, specific treatment strategies have not been well defined. We report our experience in treating these complex patients. In total, 318 men treated at our institution with chemotherapy for GCTs between 1980 and 2016 were reviewed. PMGCT was defined as clinically diagnosed mediastinal GCT with no evidence of testicular GCT (physical exam/ultrasound). We identified nine patients diagnosed with PMGCT. All patients presented with an anterior mediastinal mass and no gonadal lesion; four patients also had metastatic disease. Median age at diagnosis was 30 years (range, 14–56) and median mass size at diagnosis was 9 cm (range, 3.4–19). Eight patients had non-seminoma and one had pure seminoma. All patients received cisplatin-based chemotherapy initially. Surgical resection was performed in four patients; three patients had a complete resection and one patient was found to have an unresectable tumor. At a median follow-up of 2 years (range, 3 months–28 years) six patients had progressed. Progression-free survival was short with a median of 4.1 months from diagnosis (range 1.5–122.2 months). Five patients died at a median of 4.4 months from diagnosis. One and 5-year overall survivals were 50% and 38%, respectively. PMGCT are rare and aggressive. Our real-life Canadian experience is consistent with current literature suggesting that non-seminoma PMGCT has a poor prognosis despite prompt cisplatin-based chemotherapy followed by aggressive thoracic surgery.

## 1. Introduction

Testicular cancer accounts for 1% of malignancies among men in Canada. Among young adults aged 15–29, it is the second most common cancer accounting for 13% of cancer cases in that age group in Canada [1]. GCTs mainly arise within the testis and are broadly divided histologically to seminomas and non-seminomas. GCT’s are regarded as developmental cancers and hence can arise from extragonadal midline structures aside from the testis. Extragonadal germ cell tumors are a rare presentation of germ cell tumors (GCTs) and comprise 2–5.7% of GCT’s [2,3,4]. One common hypothesis is that these tumors originate from germ cells that migrated along the genital ridge and survived in the extragonadal environment, mainly in midline structures [5,6]. Primary mediastinal germ cell tumors (PMGCTs) are defined by a GCT of the mediastinum without a testicular primary, and account for 16–36% of extragonadal GCTs [2,7,8]. Other common sites of extragonadal GCT include the pineal gland and retroperitoneum, though the true existence of the latter is controversial [4]. PMGCTs comprise 15% of anterior mediastinal tumors in adults, and have been associated with other conditions, including Klinefelter’s syndrome and hematologic malignancies, especially megakaryoblastic leukemia [9,10,11]. Though sharing major histologic and genetic features, such as the gain in [12] isochromosome, non-seminomatous (NS), PMGCTs are more treatment-resistant and have a poorer prognosis compared to primary gonadal non-seminomas [13,14]. Hence, a diagnosis of NS-PMGCT is classified by the International Germ Cell Cancer Collaborative Group (IGCCCG) risk classification as poor prognosis, whereas the primary anatomical location of pure seminoma is not found in prognostic staging [15]. NS histology makes up the majority of PMGCT with 66–84% of cases [16,17]. NS-PMGCT have an overall survival (OS) rate of 49%, distinct from seminomatous PMGCT with an OS of 88% [17]. As these tumors are rare, data regarding the optimal treatment of PMGCTs is limited.

In this study, we summarize our experience as a regional referral center and present the institution’s experience managing these complex and challenging tumors.

## 2. Methods

Three hundred and eighteen men treated with chemotherapy at our institution between 1980 and 2016 for metastatic GCTs were identified from an electronic database and screened for a diagnosis of PMGCT. The site of origin for the primary tumor was recorded for all patients in the database. Demographic and clinical data, treatment details, and clinical outcomes, were extracted from the database and original medical records, as necessary. Descriptive statistics were used to analyze the data. This study was approved by the University of Western Ontario Research Ethics Board.

## 3. Results

Nine patients with PMGCT were identified. All patients presented with an anterior mediastinal mass proven to be GCT either pathologically or clinically (marker positive) with no evident testicular primary lesion (based on ultrasound or physical examination). Patient and tumor characteristics are summarized in Table 1. Eight patients had non-seminoma and one had pure seminoma. The median age at diagnosis was 30 years (range, 14–56 years). The most common presenting symptoms were cough (three patients), followed by hemoptysis, weight loss, fatigue and superior vena cava syndrome (two patients each). Other symptoms included: dysphagia, wheezing, upper respiratory tract infection, chest pain and hoarseness. The median mediastinal mass size at diagnosis was 9 cm (range, 3.4–19 cm). Four patients presented with metastatic disease, with three having multiple sites of metastases. Metastases sites included: lung, lymph nodes, liver and bone. Eight patients were diagnosed by mediastinal biopsy. One patient, with an elevated serum alfa-fetoprotein level, started chemotherapy treatment without histology.

The median follow-up from diagnosis was 2 years (range, 3 months–28 years). Three patients were marker negative at presentation. All patients were treated initially with cisplatin-based chemotherapy (Table 1). Either bleomycin, etoposide and cisplatin (BEP) or etoposide, ifosfamide and cisplatin (VIP) were used. Four patients responded to chemotherapy, one had stable disease, and four progressed radiographically (Table 1). Eventually, six of the nine patients had disease progression.

Aggressive thoracic surgery to completely resect residual masses was considered in four patients. Of those patients, one patient had emergent surgery for hemodynamic instability caused by tumor mass effect. At surgery viable unresectable tumor was found, and the patient subsequently died of cardiac tamponade. The other three patients had complete debulking surgery after partial or no response following chemotherapy. Surgical pathology revealed necrosis, teratoma, and gastrointestinal adenocarcinoma with sarcomatoid features considered due to malignant transformation of NS-PMGCT in one patient each.

Of the three patients that underwent complete resection, one appears to be cured, one had a long remission with recurrence after 10.1 years, and one died of treatment complications after 2 months. Apart from the cardiac tamponade noted, no major complications of thoracic surgery were noted.

Progression-free survival for NS-PMGCT was short with a median of 4.1 months (range 1.5–122.8 months). Five of the six patients that progressed did so very rapidly. The remaining patient (patient no. 9), who presumably had NS-PMGCT, did not respond initially to BEP, and hence had complete thoracic debulking surgery. That patient had malignant transformation to GI adenocarcinoma and was NED for 10.1 years before recurring and dying shortly thereafter.

One-, three- and five-year overall survival for NS-PMGCT were 50%, 38% and 38%, respectively. Three patients died rapidly within 4 months of diagnosis. Two patients were diagnosed at ages of 50 and 56 (patients nos. 1 and 3, respectively), and both had mixed GCT’s with rapidly progressive disease. 

It should be noted that the only patient with mediastinal seminoma (patient no. 6) was cured with chemotherapy alone.

## 4. Discussion

PMGCTs are uncommon. Over a 36-year period at our center, only nine cases were observed. Our experience is consistent with observations that most PMGCTs are of non-seminomatous histology, and that these are associated with a poor prognosis. However, for seminoma the occurrence of primary mediastinal disease does not appear to influence prognosis.

Standard therapy is usually based on IGCCC prognostic criteria. These criteria classify NS-PMGCTs as poor prognosis, and typically six cycles of conventional-dose cisplatin-based chemotherapy followed by complete surgical resection of residual masses are recommended. All patients in our cohort received either BEP or VIP. Although some of our patients received BEP, expert opinion currently suggests that VIP may be preferred as equally effective but associated with fewer post-surgical respiratory complications [18]. In our cohort, three of four patients having thoracic surgery had also received pre-operative bleomycin without notable pulmonary complications. One patient died after emergent surgery with unresected viable GCT due to cardiac tamponade. The other three patients had complete resections. Complete resection has been reported to be achieved in up to 86% of patients [19].

Elevated serum tumor markers are considered to be poor prognostic factors, especially β-human chorionic gonadotropin (β-HCG) [17,20]. Three NS-PMGCT patients in our cohort were marker negative at presentation. One patient appears to have been cured with chemotherapy alone, but the others rapidly progressed despite treatment. As follow-up of marker negative patients is more challenging, using novel markers can be of use. MiR-371-a-3p has been shown to outperform β-HCG and αFP with regard to sensitivity and specificity [21]. Five patients had elevated β-HCG at diagnosis, and three succumbed to progressive disease with time from diagnosis to death ranging from 2.8 to 24 months. Another patient appeared to be cured but had a lethal late recurrence at 10.1 years. Interestingly one patient had choriocarcinoma with a β-HCG of 400,000 mIU/ml with liver and lung metastasis and was cured with chemotherapy and thoracic surgery. This is unusual for mediastinal choriocarcinoma [22,23,24]. Patient 9 had malignant transformation to adenocarcinoma and sarcoma. Malignant transformation of mature teratomas into non-germ cell malignancies is a known phenomenon of GCTs [25]. Transformation can occur at presentation or in a delayed fashion and can occur to various malignancies with adenocarcinoma being one of the most common. As these tumors are chemo-resistant, prognosis is often poor [25]. Malignant transformation of PMGCT has been reported in the past [25,26,27,28,29].

Seminoma PMGCT is even more rare than NS PMGCT but harbors a much better prognosis as outlined in the IGCCCG [15]. In our cohort the single patient with seminoma PMGCT was cured with chemotherapy alone.

None of our PMGCT patients achieved marker-negative complete radiographic response. However, this is not always correlated with prognosis, as at least 25% of post-chemotherapy residual masses consist of necrotic or fibrotic tissue [19]. Indeed, in our cohort, four patients had marker-negative partial response, and three appeared cured and one had a late recurrence.

Two of our patients were diagnosed at older ages (50 and 56 years, respectively). These patients did very poorly with rapidly progressive disease despite chemotherapy and death within 4 months of diagnosis. This is consistent with previous reports suggesting older age is a poor prognostic factor [17,30]. As previously reported, patients with NS-PMGCT and visceral metastasis have an extremely poor prognosis [17,20,30]. In the current study, all three patients with visceral metastasis (liver) died within 4 months of diagnosis. One of these patients had bone metastases and died rapidly within 2.8 months of diagnosis. Indeed, bone metastasis, though very rare in GCT’s, harbor a very poor prognosis [31]. The remaining patient having choriocarcinoma (patient no.4) with initial liver and lung metastasis was cured with chemotherapy (BEP) followed by consolidative thoracic surgery.

As mentioned, an association between hematologic malignancies and PMGCT has been reported. This has been reported to occur in 1 of 17 PMGCT patients with isochromosome 12p expressed in both malignancies. This association is believed to be secondary to either an embryonic progenitor capable to differentiate to both GCT and myeloid neoplasms or alternatively leukemia derived directly from the GCT [12]. One patient in our cohort died of bone marrow suppression secondary to myelodysplastic syndrome (MDS) developed 18 months after chemotherapy completion. Obviously, we cannot determine a causative correlation between either the disease or chemotherapy and the development of MDS.

The prognosis of NS-PMGCT is much worse compared to gonadal NS [13,14,15]. Certain genomic alteration distinctions between NS-PMGCT and gonadal NS have been reported, including TP53, PIK3CA, and other cell cycle pathway genomic alterations [32]. This, along with known associations with Klinefelter’s syndrome and hematological malignancy supports the concept of a different developmental biology of NS-PMGCT compared to its gonadal counterpart.

Response rates to primary chemotherapy are lower, and cure rates with tandem high-dose chemotherapy and stem cell rescue are dismal [33]. This has led some expert opinion to recommend against use of high-dose chemotherapy for persistent or recurrent NS-PMGCT, and advocate for palliative approaches only. Intensified chemotherapy in poor prognosis NS GCT patients with inadequate tumor marker decline after their first cycle of chemotherapy appeared to improve outcomes in a randomized trial [34]. In total, 26% of the patients in this trial had NS-PMGCTs; however, subgroup analysis suggested little benefit of intensified chemotherapy in these patients. Immune check point inhibitors may benefit a subset of patients with overexpression of PD-L1, but further investigation is required [35].

Our study is limited by its small sample size, retrospective and descriptive nature. Patients were identified over a long time period at a tertiary academic center and the incidence of PMGCTs was relatively low. Certainly, treatment approaches changed over the period reviewed. However, there have been few reports on PMGCTs from Canadian expert centers.

In summary, PMGCTs are rare tumors and those with non-seminomatous histology have a poor prognosis. Primary chemotherapy followed by complete resection of residual masses is a critical component of therapy for NS-PMGCT. These tumors present a challenge for the most experienced urological and thoracic cancer teams, and we strongly recommend treatment by a multidisciplinary team at a testicular cancer expert center to optimize patient outcomes [36]. Although excellent outcomes are achieved with standard therapies for primary mediastinal seminoma, more effective drug treatment will be required to improve survival in NS-PMGCT. Due to their rarity, international collaboration will likely be required to achieve this.

## 5. Conclusions

Primary mediastinal germ cell tumors are particularly challenging to manage. Prompt aggressive chemotherapy and aggressive surgery to resect all residual disease are necessary to provide patients with an opportunity for cure. Better systemic treatment is necessary to improve cure rates. Treatment at a testicular cancer referral center is recommended.

## Figures and Tables

**Table 1:** Patient characteristics.

Pt No.	Age	Initial Histology	βhcg (mIU/mL)	αFP (ng/mL)	Metastasis	Initial Chemotherapy	Radiologic Response	Thoracic Surgery	Death	Time from Diagnosis to Death
1	50	Mixed GCT	0	0	Cervical LN×2 Lung×1	BEP×3	Progressive disease	-	Died of disease	2.8 months
2	30	Mixed GCT	300	18,000	Liver×4 Bone×2 Lung×2	BEP×3	Progressive disease	Partial resection—viable tumor	Died of disease	2.8 months
3	56	Poorly differentiated GCT	0	0	Liver×1	BEP×3	Progressive disease	-	Died of disease	4.3 months
4	42	Chorio-carcinoma	400,000	0	Liver×2 Lung×2	VIP×4	Partial response	Resection - necrosis	-	-
5	39	Mixed GCT	16	0	-	BEP×4	Partial response	-	-	-
6	27	Pure seminoma	0	0	-	VIP×4	Partial response	-	-	-
7	14	Teratoma+ Embryonal Carc­inoma	19,220	8417	-	BEP×4	Stable disease	Resection—Teratoma	Died of BM suppression	24 months
8	19	Embryonal Carcinoma	531	1670	-	BEP×4	Progressive disease		Died of disease	11 months
9	28	Non-seminoma *	287	381	-	BEP×4	Partial response	GI adenoCa+ Sarcoma	Died of disease	11.2 years

βhcg—β-human chorionic gonadotropin; GCT—Germ cell tumor; BEP—Bleomycin, Etoposide, Cisplatin; VIP—Etoposide, Ifosfamide, Cisplatin; BM—bone marrow; LN—lymph node. * No histological confirmation initially. Presence of a teratoma and non-seminoma elements most reasonable d/t clinical course and positive αFP.
